# Impact of pericardial calcification on early postoperative outcomes after pericardiectomy: a retrospective observational study

**DOI:** 10.1186/s13019-024-02842-4

**Published:** 2024-07-15

**Authors:** Soojin Lee, Juhyun Lee, Seohee Joo, You Kyeong Park, Kang Min Kim, Joon Chul Jung, Hyoung Woo Chang, Jae Hang Lee, Dong Jung Kim, Jun Sung Kim, Cheong Lim

**Affiliations:** 1 Department of Thoracic and Cardiovascular Surgery, Seoul National University Bundang Hospital, Seoul National University College of Medicine, Bundang-gu, Seongnam-si, 13620 Gyeonggi-do Republic of Korea; 2 Department of Thoracic and Cardiovascular Surgery, School of Medicine, Pusan National University, Biomedical Research Institute, Pusan National University Hospital, Busan, Republic of Korea

**Keywords:** Constrictive pericarditis, Pericardiectomy, Pericardial calcification

## Abstract

**Background:**

Owing to the lack of understanding of the clinical significance of pericardial calcification during pericardiectomy, whether pericardial calcification should be considered when determining the optimal timing for pericardiectomy is debatable. We aimed to investigate the effect of pericardial calcification on early postoperative outcomes in patients who underwent pericardiectomy for constrictive pericarditis.

**Methods:**

Altogether, 44 patients who underwent pericardiectomy for constrictive pericarditis were enrolled. After excluding three patients who underwent concurrent surgeries, a total of 41 patients were categorized into two groups based on the presence of pericardial calcification as determined by preoperative computed tomography and pathological examination. Preoperative clinical and imaging characteristics, intraoperative data, and early postoperative outcomes were compared between the two groups. A multivariable analysis was performed to identify the factors associated with postoperative complications.

**Results:**

The group with and without PC comprised 21 and 20 patients, respectively. No significant differences were observed in 30-day mortality (*n* = 1 [5%]) in the group with pericardial calcification and no mortality in the group without pericardial calcification (*p* > 0.999). Other early postoperative outcome variables did not demonstrate any significant differences between the two groups. However, the use of cardiopulmonary bypass was associated with postoperative complications (*p* < 0.009, odds ratio: 63.5, 95% confidence interval: 5.13–3400).

**Conclusions:**

Pericardial calcification did not significantly affect the postoperative outcomes after pericardiectomy. Further comprehensive studies, including those with larger sample sizes and longitudinal designs, are necessary to determine whether pericardial calcification can significantly influence the timing of surgical intervention.

## Background

The constrictive physiology observed in constrictive pericarditis results from pericardial fibrosis, which adversely affects the diastolic function. Pericardiectomy is the definitive treatment for constrictive pericarditis when constriction interferes with the cardiac output, ultimately leading to heart failure. Although pericardial calcification (PC) is frequently identified in constrictive pericarditis, its incidence ranges from 27 to 76% depending on the year of the survey and the primary cause of constrictive pericarditis within the study population [1–4].

The presence of PC has been identified as a predictive factor for unfavorable perioperative outcomes of pericardiectomy in certain studies, although contrasting findings exist in other research [1, 5–7]. Considering that calcification serves as an indicator of the chronicity and severity of the disease, surgical procedures are likely to become more extensive, potentially resulting in poorer outcomes as the disease progresses. Nevertheless, the presence of PC may be advantageous when performing pericardiectomy. Without a PC, the operation becomes technically challenging as separating layers surgically can be more difficult, leading to prolonged operative times and potentially increased perioperative bleeding. Consequently, some surgeons believe that, in cases of insignificant constriction with minimal impact on patient symptoms and hemodynamics, delaying the operation until calcification is present may be acceptable. However, a consensus regarding the surgical strategy considering the presence of PC has not been established.

We aimed to assess the influence of PC on the postoperative outcomes of patients undergoing pericardiectomy.

## Methods

### Statement of ethics

This clinical study was entirely observational and did not require registration. The research was conducted by reviewing medical records, ensuring the anonymity and non-identifiability of the patients. This study was approved by the Institutional Review Board of Seoul National University Bundang Hospital (IRB no. B-2310-856-104). The requirement for informed consent was waived owing to the retrospective study design.

### Patients and data collection

**Fig. 1:**
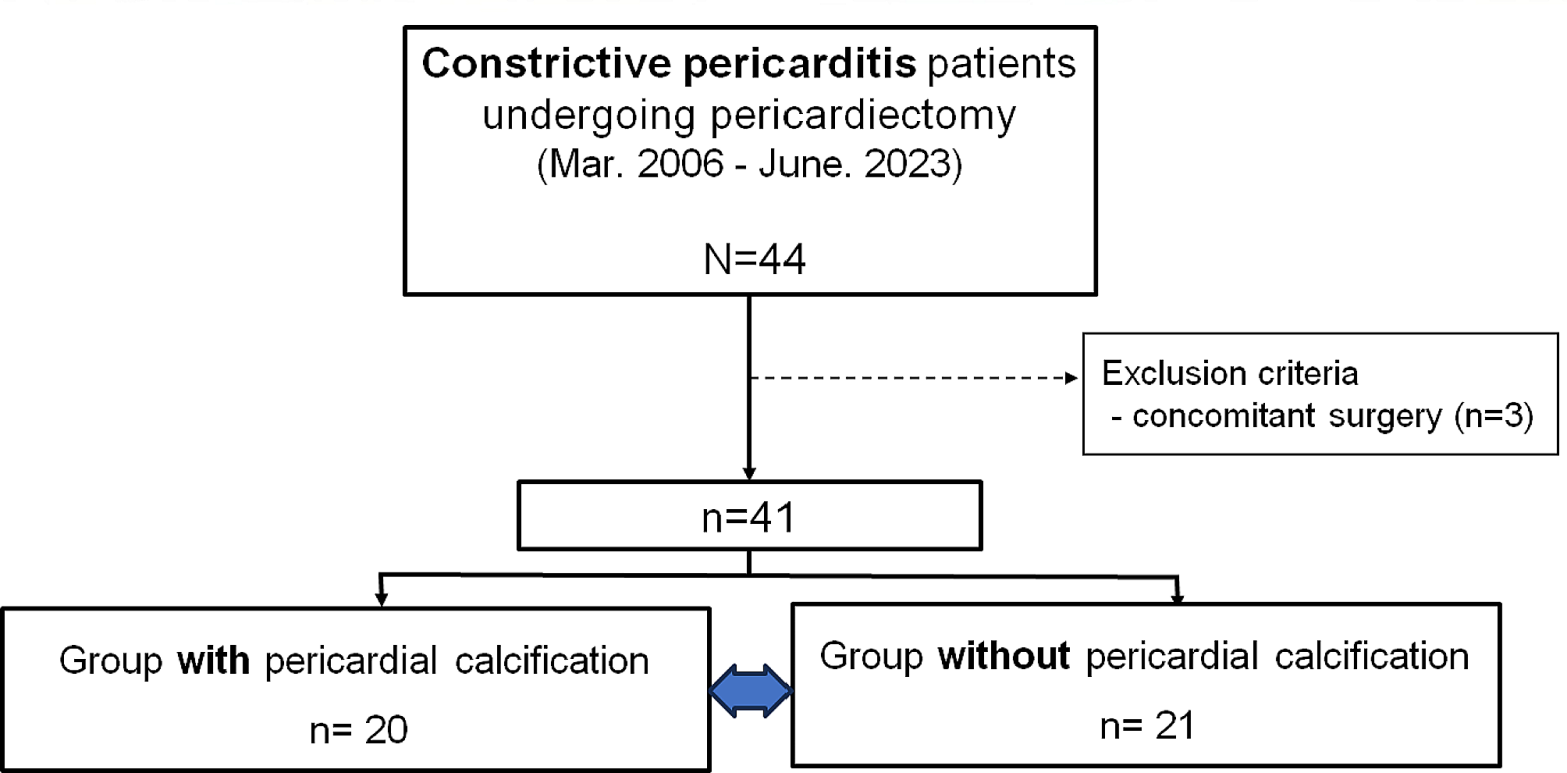
Study flowchart

The electronic medical records of 44 patients who were diagnosed with constrictive pericarditis and underwent pericardiectomy at our center between March 2006 and June 2023 were reviewed. Constrictive pericarditis was diagnostically confirmed based on echocardiographic findings and clinical symptoms. Excluding three patients who underwent concomitant procedures, a total of 41 patients were ultimately included (Fig. 1). The mean age of the patients was 58.9 ± 14.0 years, and of the total patients, 75.6% were male.

Information regarding patient characteristics, intraoperative details, and early postoperative outcomes was systematically collected. Patient characteristics included sex, age, underlying disease, history of previous cardiac surgery, etiology of constrictive pericarditis, preoperative pulmonary function test results, New York Heart Association functional classification of preoperative symptoms, duration of symptoms, left ventricular ejection fraction (LVEF), presence of PC, pleural effusion, pericardial effusion, pericardial thickness on preoperative contrast-enhanced chest computed tomography (CT), and preoperative serological findings. The diagnosis of pericardial calcification secondary to tuberculosis was based on bacteriological detection, nucleic acid analysis, and pathological examination. Central venous pressure (CVP) was measured preoperatively through catheterization following a percutaneous internal jugular vein puncture in the operating room. The intraoperative data included estimated blood loss, urgency of the operation, surgical extent categorized as partial or extended pericardiectomy, intraoperative transfusion and fluid administration, operative time, need for cardiopulmonary bypass (CPB) support, and CPB time. The early postoperative outcomes included complications during the hospital stay, total hospital stay, intensive care unit (ICU) days, ventilator days, reintubation, 30-day mortality, and readmission rate.

### Perioperative process

All the patients included in the study underwent pericardiectomy under general anesthesia. Following the standard median sternotomy, the procedure involved either partial or extended pericardiectomy. Extended pericardiectomy specifically entails the resection of the pericardium on the anterolateral side between the two phrenic nerves, the basal side over the diaphragmatic surface, over the great arteries, and from the superior vena cava to the inferior vena cava. Pericardial resection of less than these limits was classified as partial pericardiectomy [8]. CPB was selectively used based on specific criteria to avoid routine use. The decision to use CPB was guided by two primary situations: first, when vital signs became unstable, necessitating elevation of the heart to adequately release constriction, especially at the posterior atrioventricular groove, and second, when severe intraoperative bleeding rendered hemostasis challenging without decompressing the heart through CPB support. Subsequently, all the patients were admitted to the ICU before being transferred to the general ward.

#### Comparison according to the presence of PC

**Fig. 2:**
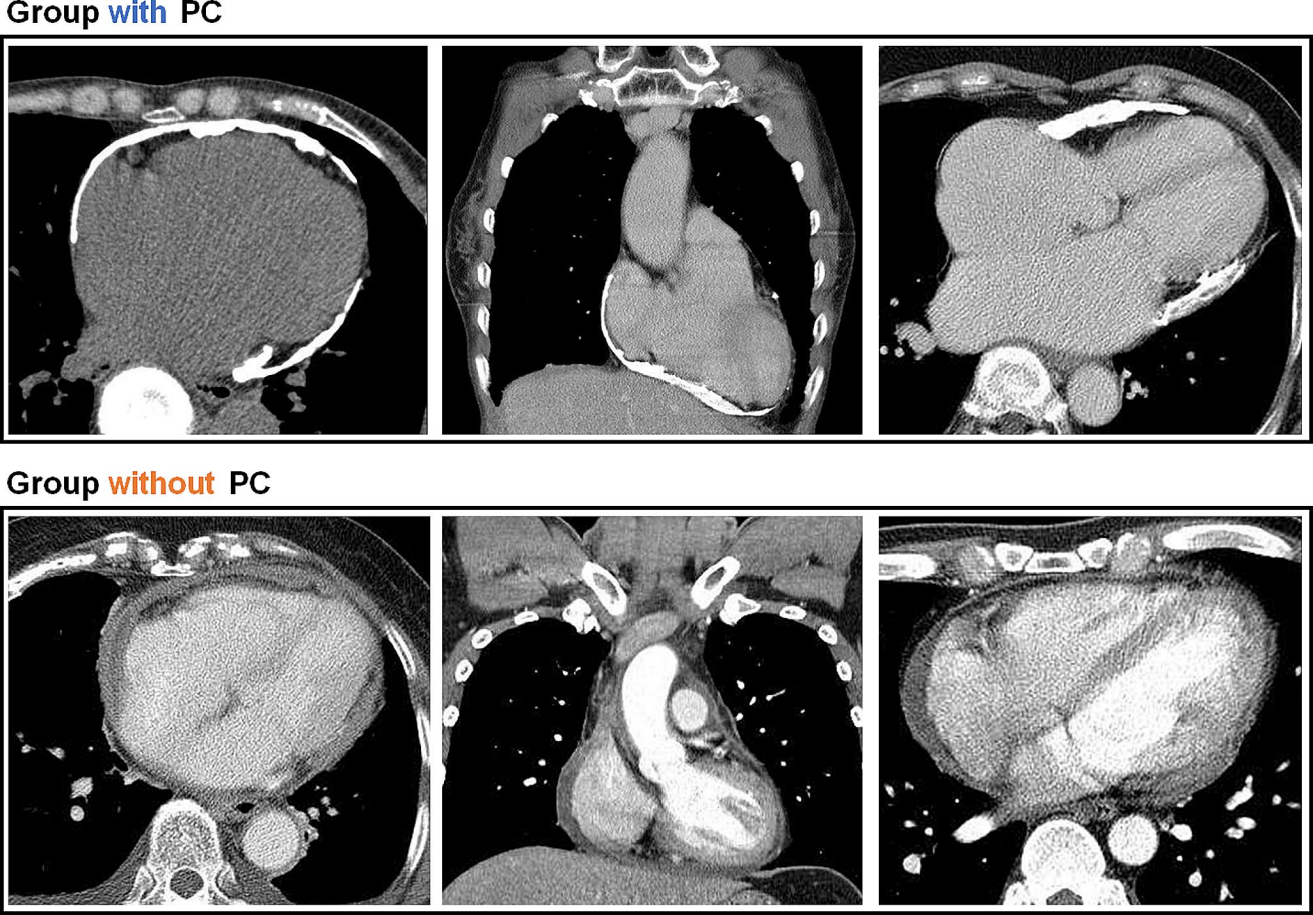
Comparative preoperative CT Imaging of patients with constrictive pericarditis, highlighting cases by presence or absence of pericardial calcification. CT, computed tomography; PC, pericardial calcification

The study participants were stratified into two groups based on the presence of PC. One group comprised patients with PC (group with PC), whereas the other group included patients without PC (group without PC). PC was confirmed by preoperative contrast-enhanced chest CT and pathological examination (Fig. 2). Preoperative patient characteristics, intraoperative data, and early postoperative outcomes were analyzed and compared between the two groups.

### Statistical analysis

Baseline characteristics between the two groups were analyzed, and intraoperative data and postoperative outcomes were compared using either an independent t-test or Wilcoxon rank-sum test for continuous variables, and the Chi-square or Fisher’s exact test for categorical variables. Kaplan-Meier curves were plotted and analyzed by using the log-rank test to compare mortality between the groups, providing further insight into the association with PC. Additionally, perioperative factors were examined based on whether patients experienced postoperative complications. Logistic regression was employed to investigate associated factors, incorporating perioperative variables with *p* < 0.1 into the multivariable analysis. Statistical significance was set at *p* < 0.05. All statistical analyses were performed using the R version 4.2.2 (R Core Team, 2020, http://cran.r-project.org).

## Results

### Baseline patient characteristics

The baseline characteristics of the patients are summarized in Table 1. The group with and without PC comprised 21 and 20 patients, respectively. In the group with PC, a significantly higher prevalence of preoperative atrial fibrillation and no significant differences in other underlying diseases, including hypertension, diabetes mellitus, chronic kidney disease, chronic obstructive pulmonary disease, or coronary artery disease, were observed. By contrast, the distribution of disease etiology demonstrated a significant difference, with idiopathic etiology being the most common in both groups, but significantly more prevalent in the group with PC. Pleural effusion, whether unilateral or bilateral, confirmed on preoperative CT, or pericardial effusion were more frequently detected in the group without PC than in the group with PC.

**Table 1:** Preoperative characteristics

			Group with PC (*n* = 20)	Group without PC (*n* = 21)	*P* value
**Age**			57.7 ± 12.5	60.0 ± 15.6	0.606
**Male, years**			13 (65.0%)	18 (85.7%)	0.238
**BMI, kg/m** ^**2**^			22.6 ± 3.6	23.7 ± 3.8	0.321
**Etiology**					0.012
	**Idiopathic**		19 (95.0%)	10 (47.6%)	
	**Postoperative**		1 (5.0%)	1 (4.8%)	
	**Tuberculosis**		0 (0.0%)	7 (33.3%)	
	**Malignancy-related**		0 (0.0%)	2 (9.5%)	
	**Mesothelioma**		0 (0.0%)	1 (4.8%)	
**Previous cardiac surgery**			3 (15.0%)	3 (14.3%)	1.000
**Underlying disease**					
	**HTN**		4 (20.0%)	4 (19.0%)	1.000
	**DM**		4 (20.0%)	2 (9.5%)	0.612
	**Atrial fibrillation**		9 (45.0%)	1 (4.8%)	0.008
	**CKD**		3 (15.0%)	1 (4.8%)	0.563
	**COPD**		2 (10.0%)	0 (0.0%)	0.447
	**CAD**		3 (15.0%)	1 (4.8%)	0.563
**PFT**	**FEV1 (%)**		78.1 ± 19.9	80.2 ± 18.9	0.750
	**FVC (%)**		79.4 ± 19.2	76.4 ± 17.7	0.628
	**DLCO**		17.5 [15.9;22.3]	20.7 [16.2;26.1]	0.260
**Preoperative symptom**					
	**NYHA class**				0.377
		I	7 (35%)	6 (28.6%)	
		II	12 (60.0%)	13 (61.9%)	
		II	0 (0.0%)	2 (9.5%)	
		IV	1 (5.0%)	0 (0.0%)	
	**Duration, month**		9.5 [1.5;18.0]	2.0 [1.0;10.0]	0.121
**LVEF, %**			56.4 ± 7.4	55.3 ± 7.1	0.608
**Pleural effusion**					0.011
	**None**		13 (65%)	5 (23.8%)	
	**Unilateral**		4 (20.0%)	4 (19.0%)	
	**Bilateral**		2 (15.0%)	12 (57.1%)	
**Pericardial effusion**			8 (40.0%)	17 (81.0%)	0.018
**Pericardial thickness, mm**			5.2 [3.9;6.5]	5.5 [3.3;8.3]	0.734
**Hepatomegaly**			6 (30.0%)	4 (19.0%)	0.651
**Hb, g/dL**			12.6 ± 1.5	13.0 ± 2.3	0.525
**Albumin, g/dL**			3.8 ± 0.5	3.7 ± 0.6	0.412

PC, pericardial calcification; BMI, body mass index; HTN, hypertension; DM, diabetes mellitus; CKD, chronic kidney disease; COPD, chronic obstructive pulmonary disease; CAD, coronary artery disease; FEV1, forced expiratory volume in the first second; FVC, forced vital capacity; DLCO, diffusing capacity of the lungs for carbon monoxide; NYHA, New York Heart Association; LVEF, left ventricular ejection fraction; Hb, hemoglobin

Values are presented as means ± standard deviations, medians [ranges], or numbers (%)

### Intraoperative data

The comparative results of intraoperative outcomes between the two groups are presented in Table 2. Although the fluid volume requirement during the operation was higher in the group without PC than in the group with PC, no significant differences in the estimated blood loss and amount of intraoperative transfusion were observed between the two groups. The group with PC exhibited a significantly longer CPB time than the group without PC (91.2 ± 28.0 min vs. 63.6 ± 23.3 min; *p* = 0.040). The CVP measurements obtained immediately before and after the operation, extent of surgery, and need for CPB support during pericardiectomy did not significantly differ between the two groups.

**Table 2:** Intraoperative data

		Group with PC (*n* = 20)	Group without PC (*n* = 21)	*P* value
**CVP**	**Pre-pericardiectomy**	20.9 ± 6.4	19.9 ± 5.2	0.580
	**Post-pericardiectomy**	10.3 ± 5.6	11.6 ± 4.8	0.432
**Surgical extent**	**Partial**	13 (65.0%)	11 (52.4%)	0.615
	**Extended**	7 (35.0%)	10 (47.6%)	
**Intraoperative volume, cc**		1477.0 ± 651.9	2528.6 ± 1376.5	0.004
**RBC transfusion (packs)**		2.0 [ 0.0; 2.5]	1.0 [ 0.0; 2.0]	0.145
**EBL, cc**		800.0 [300.0;1200.0]	750.0 [300.0;1250.0]	0.953
**CPB support**		10 (50.0%)	8 (38.1%)	0.651
**CPB time, min**		91.2 ± 28.0	63.6 ± 23.3	0.040
**Operative time, min**		220.2 ± 62.9	199.0 ± 76.1	0.338

PC, pericardial calcification; CVP, central venous pressure; RBC, red blood cell; EBL, estimated blood loss; CPB, cardiopulmonary bypass

Values are presented as means ± standard deviations, medians [ranges], or numbers (%)

### Postoperative outcomes and complications

**Fig. 3:**
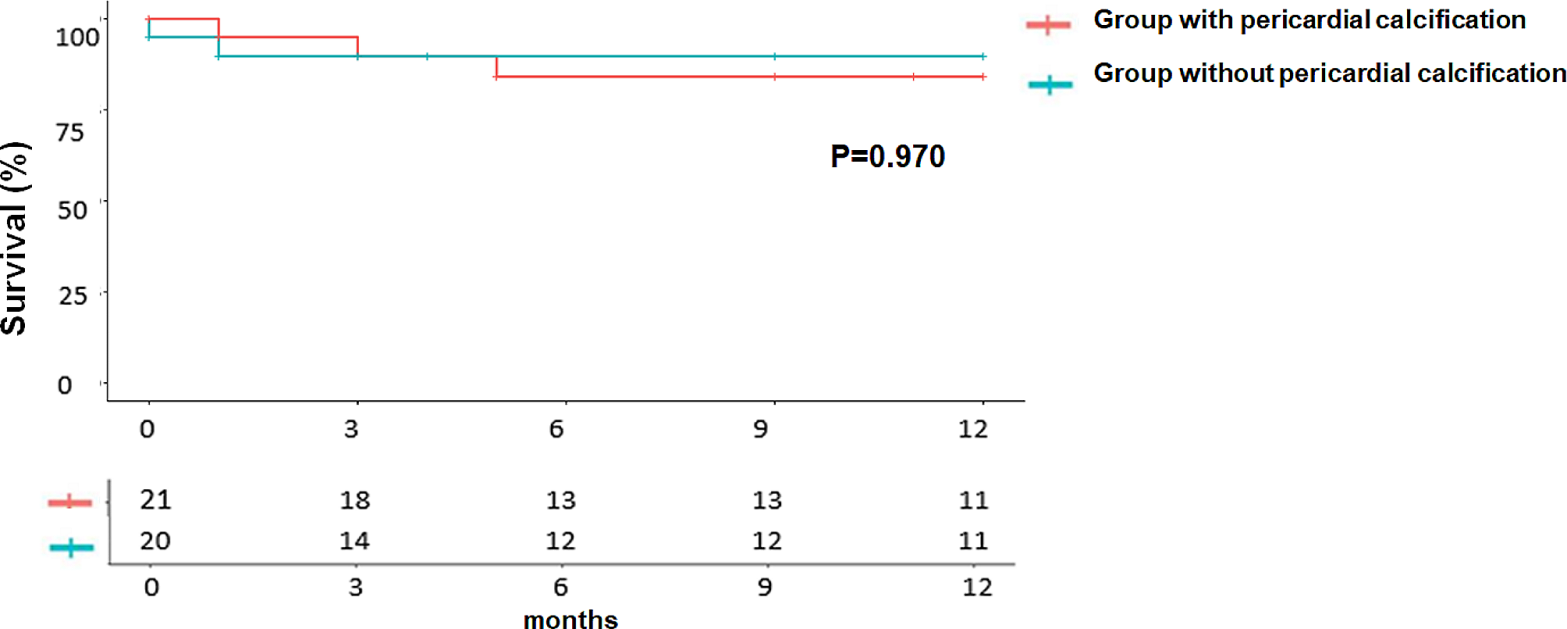
One-year survival among patients with constrictive pericarditis, stratified by presence or absence of pericardial calcification

The comparative results of the postoperative outcomes between the two groups are summarized in Table 3. No significant differences in mortality or postoperative complications were noted between the two groups. The Kaplan–Meier curves comparing the mortality of the two groups are illustrated in Fig. 3.

**Table 3:** Postoperative outcomes

		Group with PC (*n* = 20)	Group without PC (*n* = 21)	*P* value
**Mortality**	**< 30 days**	1 (5.0%)	0 (0.0%)	> 0.999
	**< 1 year**	9 (15.9%)	10 (10.3%)	0.790
**Complication**	**Total**	7 (35.0%)	7 (33.0%)	> 0.999
**Hospital stay, day**		9.5 [6.5;13.5]	9.0 [8.0;14.0]	0.783
**ICU stay, day**		1.5 [1.0; 3.0]	1.0 [1.0; 3.0]	0.408
**Ventilator day**		0.0 [0.0; 1.0]	0.0 [0.0; 1.0]	0.929
**Readmission**		5 (25.0%)	3 (14.3%)	0.638
**Postoperative NYHA class**				0.214
	**I**	17 (85.0%)	21 (100%)	
	**II**	3 (15.0%)	0 (0.0%)	
**Dyspnea improvement**		10 (50.0%)	15 (71.4%)	0.278
**Postoperative echocardiography**				
	**LVEF, %**	58.0 [53.3;60.5]	60.0 [55.3;62.0]	0.272
	**LVEF improvement, %**	2.5 [-2.5; 6.5]	5.0 [ 0.3; 7.0]	0.290
	**Improved constrictive physiology**	15 (75.0%)	20 (95.2%)	0.164
	**Remnant constrictive physiology**	9 (45.0%)	8 (38.1%)	0.895
	**Increased pericardial thickness**	7 (35.0%)	8 (38.1%)	> 0.999
	**Septal bouncing**	17 (85.0%)	16 (76.2%)	0.751
	**Ventricular interdependency**	5 (27.8%)	3 (14.3%)	0.521
	**IVC plethora**	9 (45.0%)	8 (40.0%)	> 0.999
	**MV/TV inflow**	4 (22.2%)	5 (23.8%)	> 0.999

PC, pericardial calcification; ICU, intensive care unit; NYHA, New York Heart Association; LVEF, left ventricular ejection fraction; IVC, inferior vena cava; MV, mitral valve; TV, tricuspid valve

Values are presented as medians (ranges) or numbers (%)

Of 41 patients, 14 (34.1%) experienced postoperative complications. The most common complications were new-onset atrial fibrillation (*n* = 3, 7.3%) and postoperative bleeding requiring reoperation for hemostasis (*n* = 3, 7.3%). Low cardiac output syndrome and acute respiratory distress syndrome occurred in two patients (4.8%). Mediastinitis, stroke, and sinus pauses were identified in one patient in each group (2.4%). One patient underwent reoperation because of persistent dyspnea of NYHA class III with significant constrictive physiology including persistent septal bouncing, ventricular interdependency and similar thickness of pericardium compared with previous finding, as indicated by postoperative transthoracic echocardiography (TTE) and chest CT on postoperative day 4. (Table 4).

**Table 4:** In-hospital postoperative complications

			Group with PC (*n* = 20)	Group without PC (*n* = 21)	*P* value
**Complication**	**Total**	**14 (34%)**	7 (35.0%)	7 (33.0%)	> 0.999
	New atrial fibrillation	3 (7.3%)	2 (10.0%)	1 (4.7%)	
	Postoperative bleeding	3 (7.3%)	1 (5.0%)	2 (9.5%)	
	LCOS	2 (4.8%)	1(5.0%)	1 (4.7%)	
	ARDS	2 (4.8%)	1 (5.0%)	1 (4.7%)	
	Mediastinitis	1 (2.4%)	0 (0.0%)	1 (4.7%)	
	Stroke	1 (2.4%)	0 (0.0%)	1 (4.7%)	
	Sinus pause	1 (2.4%)	1 (5.0%)	0 (0.0%)	
	Redo-pericardiectomy	1 (2.4%)	1 (5.0%)	0 (0.0%)	

ARDS, acute respiratory distress syndrome; LCOS, low cardiac output syndrome

### Postoperative changes in symptoms and echocardiographic findings

**Fig. 4:**
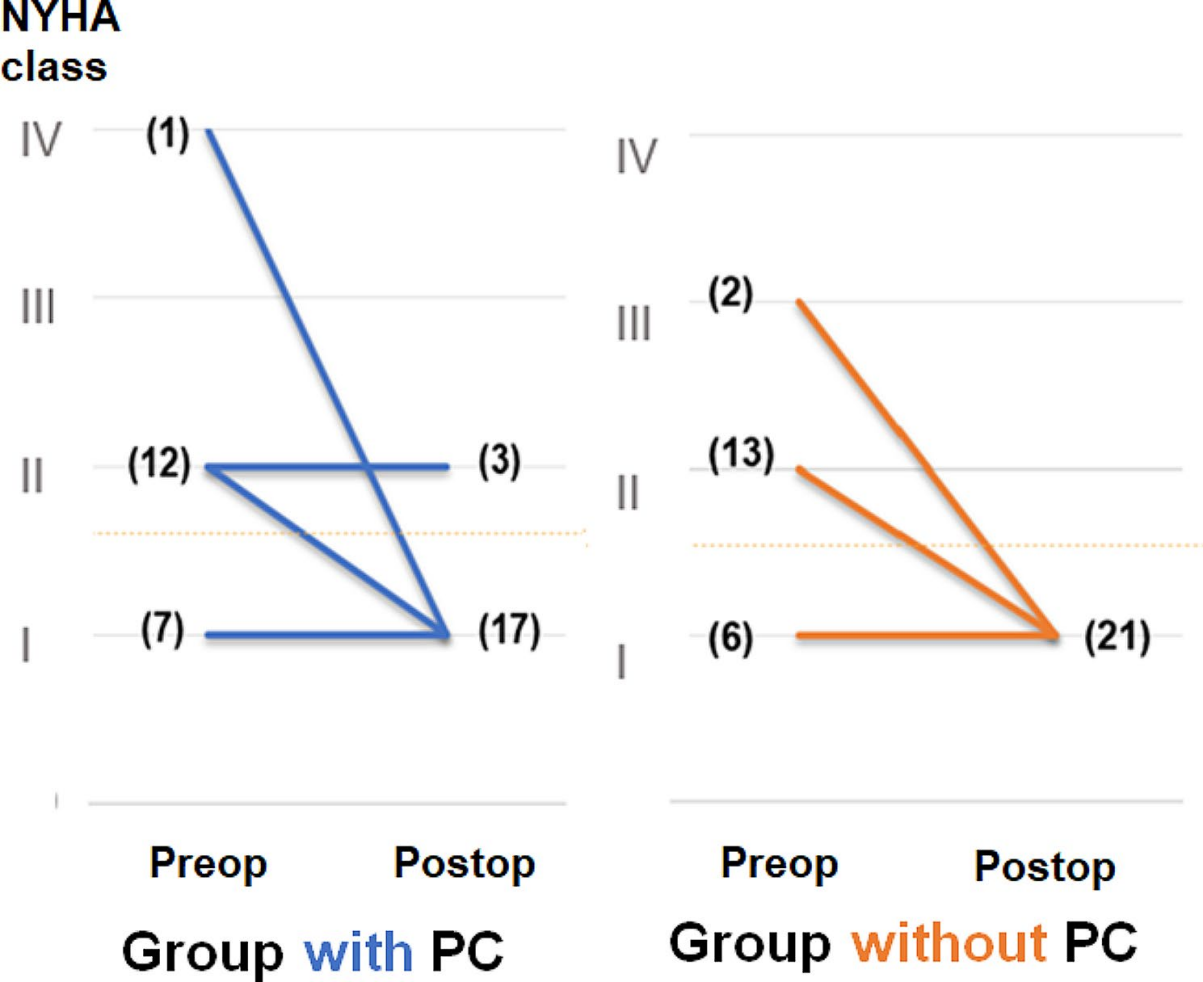
Comparison of symptom improvement after pericardiectomy, according to the presence of pericardial calcification. PC, pericardial calcification; NYHA, New York Heart Association

In both groups, the symptoms improved postoperatively (Fig. 4). However, no significant differences in the improvement of postoperative symptoms and functional markers measured by postoperative TTE were observed between the two groups (Table 3).

### Factors associated with postoperative complications

The association between postoperative complications and perioperative factors was analyzed using logistic regression. Univariable analysis revealed that postoperative complications were significantly associated with age, preoperative LVEF, preoperative hypoalbuminemia, and CPB support (*p* < 0.05 for all four variables). Ultimately, following the inclusion of significant factors from the initial analysis, multivariable analysis demonstrated a significant association between postoperative complications and CPB support (*p* = 0.009; odds ratio, 63.5; 95% confidence interval, 5.13–3400) (Table 5).

**Table 5:** Logistic regression analysis of factors associated with postoperative complications

Variables	Univariable analysis		Multivariable analysis	
	OR (95% CI)	P value	OR (95% CI)	*P* value
**Male**	1.473 (0.335–7.869)	0.620		
**Age**	1.057 (1.005–1.121)	0.042	1.07 (0.98–1.2)	0.181
**HTN**	0.193 (0.009–1.269)	0.145		
**DM**	1.916 (0.313–11.808)	0.465		
**Arrythmia**	1.212 (0.262–5.210)	0.797		
**COPD**	1.785 (0.067–47.550)	0.689		
**Preoperative CVP**	1.074 (0.954–1.231)	0.262		
**Etiology**	0.802 (0.472–1.230)	0.349		
**Redo-pericardiectomy**	0.846 (0.107–4.978)	0.858		
**Dyspnea improvement**	1.097 (0.410–2.872)	0.851		
**Pleural effusion**	1.318 (0.66–2.747)	0.448		
**Pericardial effusion**	0.605 (0.162–2.232)	0.448		
**Pericardial calcification**	0.875 (0.239–3.142)	0.837	0.3 (0.02–2.89)	0.350
**Pericardial thickness**	0.762 (0.533–1.004)	0.090	0.57 (0.24–0.99)	0.093
**Preoperative LVEF**	0.903 (0.805–9.946)	0.049	0.86 (0.68–1.02)	0.121
**Hypoalbuminemia**	12.5 (1.736–256.05)	0.029	9.9 (0.69–326)	0.128
**Intraoperative fluid volume**	0.999 (0.999-1.000)	0.944		
**Operative time**	1.009 (0.999–1.020)	0.075		
**CPB support**	7.464 (1.899–34.98)	0.006	63.5 (5.13–3400)	0.009

OR, odds ratio; CI, confidence interval; HTN, hypertension; DM, diabetes mellitus; COPD, chronic obstructive pulmonary disease; CVP, central venous pressure; LVEF, left ventricular ejection fraction; CPB, cardiopulmonary bypass

## Discussion

The presence of pericardial calcification was found to have no significant correlation with early postoperative outcomes. Similar findings have been observed in previous studies; however, those studies either focused on patients with constrictive pericarditis who did not undergo surgical treatment or were limited to analyzing surgical patients with respect to mid-term postoperative outcomes [9, 10]. Several studies have identified PC as a negative postoperative prognostic factor, attributing it to the chronic nature of the disease and its associated poorer outcomes [7]. Generally, PC develops because of repetitive inflammatory responses to noxious stimuli in the mesoderm of the pericardium [11]. Consequently, constrictive pericarditis with PC may exhibit a more chronic course than cases without calcification. However, it is crucial to acknowledge that pericardial calcification can be detected as a sequela of constrictive pericarditis, regardless of whether the tuberculosis is chronic or of recent onset [12]. In such cases, the association between disease progression and calcification may not be apparent, particularly when the predominant etiology within the study population is considered [4]. The postoperative outcomes of pericardiectomy in patients with constrictive pericarditis are influenced by a combination of factors including the extent of surgical intervention, preoperative comorbidities, and constrictive pericarditis categorized by etiology. Therefore, a comprehensive interpretation is imperative to draw conclusions regarding postoperative prognosis based on the presence of PC.

On reviewing our 16-year experience with pericardiectomy for constrictive pericarditis, we observed that the presence of PC did not consistently result in poor outcomes. The similar operative times, regardless of the presence of PC, nullified our initial hypothesis. However, whether PC is a crucial guide in surgical dissection, aiding in the differentiation of layers during pericardiectomy, is controversial. Of three highly experienced cardiac surgeons, two favored the consideration of PC, believing that the presence of PC should be one of the factors that should be considered during the decision for surgical timing, whereas the other surgeon disagreed. The latter asserted that the decision to perform pericardiectomy should be based solely on the severity of the patient’s symptoms and evident constrictive physiology observed on echocardiography, regardless of calcification. Although the majority of the surgeons in our study believed that PC might serve as a procedural guide during the operation, potentially reducing operative time, the findings indicated that its presence did not significantly reduce the overall operative time. We hypothesize that PC might have served as a target, streamlining the dissection process but simultaneously extending the operative time by prompting surgeons to exert additional effort to completely remove the calcified tissue. The fact that most resections were performed during CPB support, and the noticeably extended CPB duration in the group with PC, reinforced our explanation. The clearer the target, the more determined the surgeons were to achieve a thorough resection. This may have contributed to the similar operative times between the two groups. However, these explanations may not comprehensively account for our observations, and the proposed theoretical framework represents only one of several speculations arising from the retrospective analysis of our results. Considering the inherent constraints of a retrospective study and the inclusion of multiple surgeons with diverse pericardiectomy strategies, further investigation is crucial to obtain more precise insights. A meticulous examination, including the segregation of results by individual surgeons, is necessary to unravel the intricate factors influencing CPB time in the context of pericardiectomy.

We also discovered that CPB support is associated with early postoperative complications. In the prior multivariable analysis, the requirement for cardiopulmonary bypass (hazard ratio [HR]: 21.2, *p* = 0.02) was identified as a predictor of 30-day mortality, which is consistent with our findings [13]. Considering that the prevalent complications are new-onset atrial fibrillation and postoperative bleeding, this association is theorized to originate from the production of cytokines during the sequential systemic inflammatory response triggered by CPB, potentially serving as a catalyst for atrial fibrillation [14]. Additionally, the correlation between CPB use and postoperative complications might be explained by the bleeding tendencies induced by intravenous heparin administration during CPB and platelet dysfunction associated with CPB support [15]. As we have already demonstrated in the perioperative process section of the Methods, the CPB selection strategy remained consistent between the groups with and without PC. While CPB may affect postoperative complications, it is important to acknowledge that the statistical associations identified through regression analysis do not inherently imply causation [16]. Therefore, further investigations, including longitudinal studies, are warranted to explore and establish causality in the relationship between CPB use and postoperative complications.

Our unexpected and novel findings regarding the clinical impact of PC on postoperative outcomes may assist cardiac surgeons in making decisions regarding pericardiectomy, irrespective of the presence or absence of PC. Because pericardiectomy is associated with relatively high postoperative mortality and morbidity [13] the evaluation of preoperative risks and determination of surgical timing are crucial. PC is a common feature of constrictive pericarditis; however, its effect on postoperative outcomes has rarely been explored [17]. Despite the seemingly small sample size in our study, pericardiectomy is less common than other cardiac surgeries, such as coronary artery bypass grafting or valve procedures. This is primarily attributed to the lower incidence of constrictive pericarditis compared to that of other cardiac diseases [18]. To the best of our knowledge, our study is the first to examine the clinical importance of PC, taking into account both preoperative and intraoperative factors. It utilizes the largest sample size to date, as the research was conducted at a high-volume center in South Korea.

Our study’s limitations include a relatively small sample size and the fact that it is a single-center retrospective study. Therefore, careful interpretation is necessary as the statistical power derived from a small sample size is limited. Conducting additional research through a multi-center study could overcome the issues related to the limited sample size typical of rare diseases and surgical cases. Furthermore, this study, based on patient data spanning 16 years, suggests the potential influence of long-term research effects on the outcomes. Over the course of 16 years, advancements in surgical techniques and equipment, alongside the accumulation of surgical experience, may have occurred. Additionally, there may have been changes in intensive care unit treatment strategies. These factors represent limitations of the study and underscore the necessity for further multicenter investigations.

Further studies that consider the severity and distribution of calcification may more precisely elucidate the clinical significance of PC. The myocardium surrounded by calcification may be directly affected, and the symptoms and degree of diastolic dysfunction may vary depending on the part of the heart involved in the calcification. In our study, we considered the pericardial calcification and calcification thicknesses have no association with postoperative complications. However, this study had limitations in assessing the location of calcification and in quantifying calcium using a calcium scoring system on CT. Future investigations are required to explore the influence of PC by examining both its location and severity using a calcium scoring system.

## Conclusion

The clinical influence of PC on postoperative outcomes was not significant after pericardiectomy. Consequently, when deciding the optimal timing for pericardiectomy, PC may not be considered a crucial factor. Instead, providing prompt surgical intervention should be prioritized, considering the severity of the patient’s symptoms and the evident constrictive physiological markers observed on echocardiography.

## Data Availability

No datasets were generated or analysed during the current study.

## Abbreviations

CT: Computed tomography
LVEF: Left ventricular ejection fraction
CVP: Central venous pressure
CPB: Cardiopulmonary bypass
ICU: Intensive care unit
TTE: Transthoracic echocardiography
